# Supplementary material for: P-TRAP: a Panicle Trait Phenotyping tool
Source: BMC Plant Biol. 2013 Aug 29;13:122. doi: 10.1186/1471-2229-13-122 (PMC3848748; doi:10.1186/1471-2229-13-122)
Supplement: Additional file 7 — P-TRAP source code. The Netbeans project that contains the source code of the application. [file 1471-2229-13-122-S7.zip › javadoc.zip!/javadoc/org-bioalg-mathprocessor/index-all.html]

Index (MathProcessor)


JavaScript is disabled on your browser.


- Package
- Class
- Tree
- Deprecated
- Index
- Help

*org.bioalg.mathprocessor 1.0*

- Prev
- Next

- Frames
- No Frames

- All Classes

A B E G I M O P R S V 


## A

angle(Point, Point) - Static method in class org.bioalg.mathprocessor.BasicMath
:   The angel between two points.


## B

BasicMath - Class in org.bioalg.mathprocessor
:   The Basic mathematical operations used in image processing in report
    calculation.

BasicMath() - Constructor for class org.bioalg.mathprocessor.BasicMath


## E

eculidian(Point, Point) - Static method in class org.bioalg.mathprocessor.BasicMath
:   The Euclidian distance between two points.


## G

getSlop(Point, Point) - Static method in class org.bioalg.mathprocessor.BasicMath
:   Get the slop between two points

getSlopinv(Point, Point) - Static method in class org.bioalg.mathprocessor.BasicMath


## I

isBoolean(String) - Static method in class org.bioalg.mathprocessor.BasicMath
:   Return true if the input value is boolean.

isdouble(String) - Static method in class org.bioalg.mathprocessor.BasicMath
:   Return true if the input value is double

isHorizontal(Point, Point) - Static method in class org.bioalg.mathprocessor.BasicMath
:   Check if two points are horizontally colinear

isInteger(String) - Static method in class org.bioalg.mathprocessor.BasicMath
:   Return true if the input value is integer.

IsPerpendicular(Point, Point, Point, Point) - Static method in class org.bioalg.mathprocessor.BasicMath
:   check if two lines are perpendicular.

isVertical(Point, Point) - Static method in class org.bioalg.mathprocessor.BasicMath
:   Check if two points are horizontally colinear


## M

max(double[][]) - Static method in class org.bioalg.mathprocessor.BasicMath
:   The maximum of a matrix.

mean(int[]) - Static method in class org.bioalg.mathprocessor.BasicMath
:   Return the mean of an integer vector

mean(int[][]) - Static method in class org.bioalg.mathprocessor.BasicMath
:   Return the mean of a matrix


## O

org.bioalg.mathprocessor - package org.bioalg.mathprocessor


## P

pixels2centimeter(double) - Static method in class org.bioalg.mathprocessor.BasicMath
:   Uses the application options to map pixels to centimeters

pixels2Inches(double) - Static method in class org.bioalg.mathprocessor.BasicMath


pointInLine(Point, Point, Point) - Static method in class org.bioalg.mathprocessor.BasicMath
:   Return true if a given point is in a line, false otherwise.

pointToLineDistance(Point, Point, Point) - Static method in class org.bioalg.mathprocessor.BasicMath
:   The distance from a point to a line.


## R

roundTO5Decimals(double) - Static method in class org.bioalg.mathprocessor.BasicMath
:   Round to 5 decimals

roundTO5Decimals2(double) - Static method in class org.bioalg.mathprocessor.BasicMath


roundTwoDecimals(double) - Static method in class org.bioalg.mathprocessor.BasicMath
:   Round a double value to two ddecimals

roundTwoDecimals2(double) - Static method in class org.bioalg.mathprocessor.BasicMath


## S

STD(int[], double) - Static method in class org.bioalg.mathprocessor.BasicMath
:   Return the standard deviation of vector given its mean

STD(int[][], double) - Static method in class org.bioalg.mathprocessor.BasicMath
:   Return the standard deviation of an integer matrix.

sum(int[]) - Static method in class org.bioalg.mathprocessor.BasicMath
:   The sum of an integer vector

sum(double[]) - Static method in class org.bioalg.mathprocessor.BasicMath
:   The sum of double vector.

sum(int[][]) - Static method in class org.bioalg.mathprocessor.BasicMath
:   The sum of an integer matrix

swapXY(Point) - Static method in class org.bioalg.mathprocessor.BasicMath
:   Swap a point's XY


## V

variance(double[]) - Static method in class org.bioalg.mathprocessor.BasicMath
:   The variance of a double array.

varinace(double[][]) - Static method in class org.bioalg.mathprocessor.BasicMath
:   calculate the variance of a matrix

A B E G I M O P R S V

- Package
- Class
- Tree
- Deprecated
- Index
- Help

*org.bioalg.mathprocessor 1.0*

- Prev
- Next

- Frames
- No Frames

- All Classes
